# Supplementary material for: The Frequency and Healthfulness of Food and Beverage Advertising in Movie Theatres: A Pilot Study Conducted in the United States and Canada
Source: Nutrients. 2020 Apr 28;12(5):1253. doi: 10.3390/nu12051253 (PMC7282003; doi:10.3390/nu12051253)
Supplement: Supplementary file 1 [file nutrients-12-01253-s001.pdf]

Title: The frequency and healthfulness of food and beverage advertising in movie theatres: A pilot study conducted in the United States and Canada:

Authors: Wong, S., Pauzé, E., Hatoum, F., Potvin Kent, M.

Table S1: Frequency and rating of movies seen in Ontario and Virginia during data collection period in Feb-May 2019

Title	Rating ON / VA	Ontario (ON) N = 28	Virginia (VA) N = 36
		n (%)	n (%)
A Dog's Journey	PG / PG	2	2
A Dog's Way Home	PG / PG	1	1
Aladdin	PG / PG	1	1
Avengers: Endgame	PG / PG13	1	0
Biggest Little Farm	-- / PG	0	3
Captain Marvel	PG / PG13	1	0
Detective Pikachu	PG / PG	4	2
Disney Penguins	G / --	1	0
Dumbo	PG / PG	1	7
How to Train Your Dragon 3	PG / PG	5	7
Lego Movie 2	PG / PG	3	5
Mary Poppins Returns	PG / PG	0	1
Mia and the White Lion	G / --	1	0
Missing Link	PG / PG	1	0
Shazam!	PG / PG13	2	0
Smallfoot	PG / PG	1	0
The Kid Who Would Be King	PG / PG	1	1
Ugly Dolls	PG / PG	0	1
Wonder Park	PG / PG	1	5
Wreck It Ralph 2	PG / PG	1	0

Note: Some movies were only played in Ontario (Mia and the White Lion) and some only played in Virginia (Biggest Little Farm). Generally, most movies played in both Ontario and Virginia. Movies rated PG13 in the VA were not considered for inclusion in the study.
